# Changes in family socio-economic status as predictors of self-efficacy in 13-year-old Polish adolescents

**DOI:** 10.1007/s00038-013-0458-1

**Published:** 2013-04-02

**Authors:** Joanna Mazur, Agnieszka Malkowska-Szkutnik, Izabela Tabak

**Affiliations:** Department of Child and Adolescent Health, Institute of Mother and Child, Warsaw, Poland

**Keywords:** Self-efficacy, Educational mobility, Family affluence, Mediating effect, Quality of life

## Abstract

**Objectives:**

The aim of this study is to determine the impact that raised mother’s education and a relative change in family affluence might have on adolescent general self-efficacy (GSE).

**Methods:**

Data on 600 children born in Poland in January 1995 and their families were used. Data from early childhood and adolescence (2008) were considered and the change between these two periods was determined.

**Results:**

Family affluence increased in 37.3 % of families with mothers, who had raised their education level (12.6 % of the sample), in comparison to 26.8 % in the group with no change, *p* < 0.001. The average GSE scores in those groups were 73.4 and 68.1, respectively, *p* < 0.001. In the best linear regression model adjusted for gender, the independent predictors of GSE turned out to be mother’s education change and the family’s current affluence.

**Conclusions:**

Raised mother’s education level may encourage building up developmental assets in older children. Based on the structural model, where self-efficacy is the mediator of the relationship between socio-economic status change and the quality of life (KIDSCREEN-10) these results may be of importance in further research.

## Introduction

In research concerning adolescent health, particular attention was focused currently on health-promoting personal characteristics (Morgan and Zigilio 2007). Self-efficacy defined as “one’s capabilities to organize and execute the courses of action required to produce given attainments” (Bandura 1997) could be considered to be an important internal resource and health determinant. Self-efficacy contributed to academic achievements; pro-social behaviours; and in preventing some mental health problems in adolescents (Bandura et al. 1999). It was considered to be one of the most important determinants of health behaviour and was applied in various models of changes in health behaviour (Masten et al. 1990; Luszczynska et al. 2011).

Self-efficacy could be associated with psychological aspects of health (e.g. subjective well-being; feeling happy; and perceived social support) (Natvig et al. 2003). Bandura described four sources of self-efficacy: performance accomplishments; verbal persuasion; emotional arousal; and vicarious experiences (Bandura 1976). The sources could be found in family, school and peer environments and, also, amongst macro-social determinants (van Dinther et al. 2011).

Until now, not much attention was paid to the impact of family socioeconomic status (SES) on personal competences conducive to adolescent health. Although the relationship between personal characteristics and health was a subject of research in health psychology, the SES’ impact on health was included in sociological research. Only a few studies attempted to combine the integrated psycho-sociological model with research on social inequalities in the life course. Lundberg (1997) presented a conceptual model which linked childhood conditions; sense of coherence; adult social class; and adult health. He concluded that the sense of coherence might have been a factor involved in shaping of social inequalities in health.

Most studies, which analysed social health inequalities, from the life course perspective, dealt with the impact of family SES in early childhood on adult health. There were less frequent attempts to assess the impact of early childhood factors on the health of older children and adolescents. Regardless of the target group, the impact of living conditions, in childhood, on later life ought to be discussed along with a number of indirect links with health (Kuh et al. 2003). Living conditions, in the first years of life, might have an impact on diseases and developmental disorders that occurred later in life and, subsequently, could influence health (Graham and Power 2004). However, living conditions might act, also, as reinforcement; shaping positive health behaviours and building up external and internal health-related resources such as self-efficacy.

Researchers, adopting the life course approach, claimed that it ought not to be linked simply with longitudinal studies. The life course approach focused, also, on developing theoretical models; these would help to understand biological, psychosocial and behavioural health determinants in a dynamic setting. The “critical development periods” model and the “cumulative risk exposure” model were amongst the most frequently quoted (Graham 2002). The “change” model, used in this study, was the third most commonly applied model (Cohen et al. 2010; Walsemann et al. 2008), in which the change in family SES was measured usually by devising social mobility patterns or by creating a cumulated index including information from different stages of life (Luo and Waite 2005).

Changes, in the family SES, might indicate a number of parental features and behaviours which shaped adolescent personalities and influenced their health and development. However, until now, educational achievements, instead of health indicators, were used more frequently as outcome measures. In particular, there was a need to investigate the impact of an increase in the parental education level and family affluence on child health and psychosocial development. In younger children, the impact could be negative because the large amount of time, dedicated by the parents to further education or work, might disrupt their relationship with the child. However, a positive relationship, between the parents and the child, depended both on the amount of time spent with the child and on the quality of the relationship (Desha et al. 2011). In older children, a positive impact was more likely, considering the possible improvement of living conditions and general cultural capital of the family, as well as the positive patterns and skills (perseverance; diligence; clearly defined life goals) which parents passed on to children (Graziano et al. 2009). Researchers called this phenomenon “the independent education effect”.

This study aimed to determine the impact which a raised mother’s education level and a relative change in family affluence might have on adolescent self-efficacy. An attempt was made, also, to verify a more complex model. It took into account the impact of self-efficacy on general adolescent well-being, and included changes in the family SES as compared to the early childhood period.

## Methods

### Design and participants

**Fig. 1:**
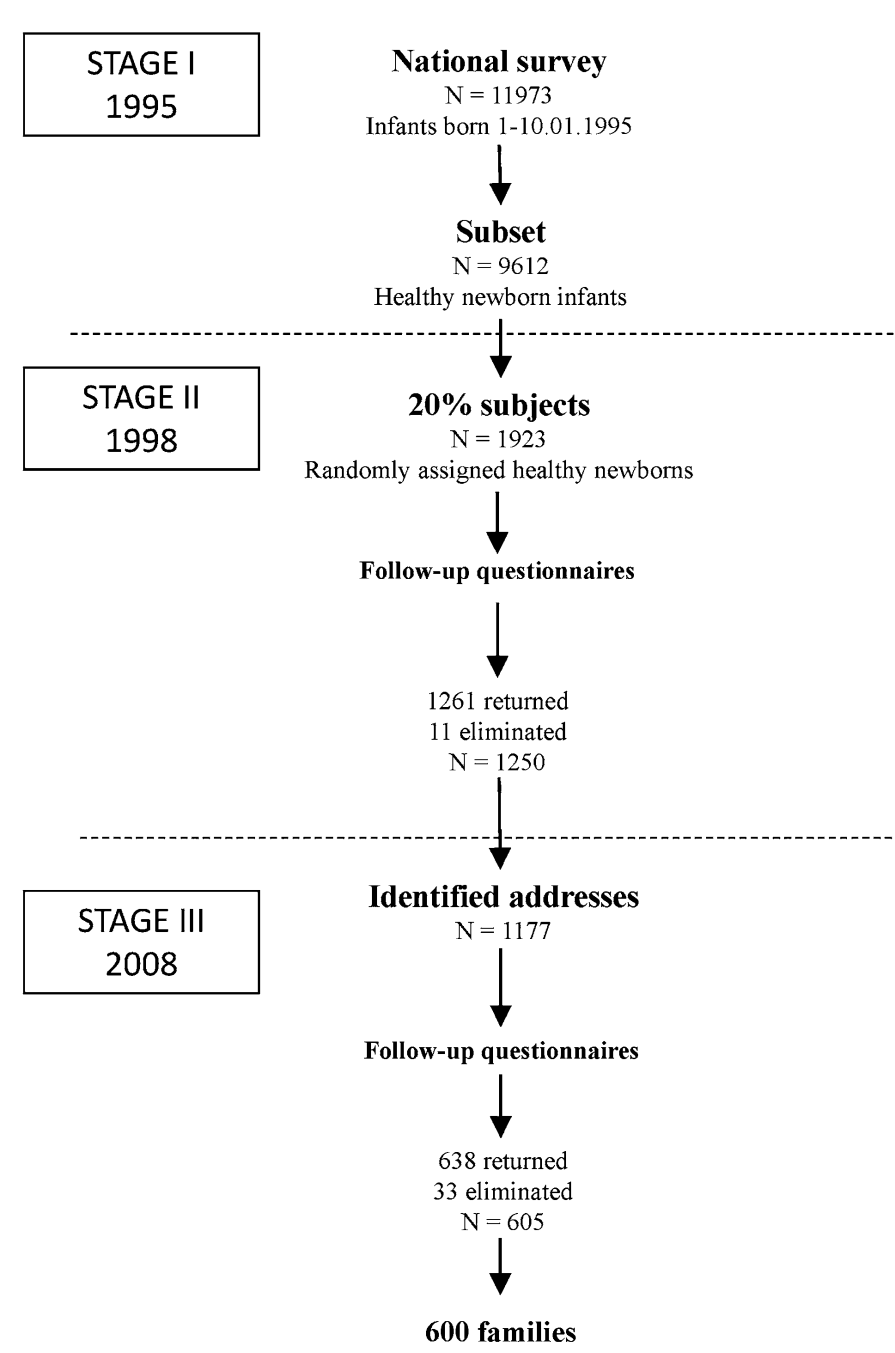
Flowchart of sample selection from the Polish cohort study (1995–2008)

Data were used from a prospective cohort study with the main objective of examining the impact of early childhood factors (mostly nutritional) on body weight disorders in adolescents. Figure 1 illustrates this study’s three stages. The first two stages were funded by a governmental health policy programme (Mikiel-Kostyra et al. 2002), whilst the third one was an independent project (Jodkowska et al. 2010). In the first phase, hospital staff carried out a survey in 427 maternity wards and included all children born in Poland between 1 and 10 January 1995. Three years later (January 1998), a subset of 9,612 healthy children was selected. The following inclusion criteria were applied: gestational age ≥37 week; birth weight ≥2,500 g; lack of neonatal problems, and discharge at 2–15 days after birth. Then, by systematic sampling of every fifth case (20 %), 1,923 children were selected randomly. A postal survey was conducted with a response rate of 65.0 %. In the third phase, 10 years later, 94.2 % of the families who took part in the second stage, were identified, and separate questionnaires for parents and children were issued. The response rate to the third stage was 51.4 %. Six hundred 13-year-old children formed the cohort of the current analysis.

### Dependent variables

The main dependent variable was the average self-efficacy score which was measured by using the GSE (General Self-Efficacy) questionnaire designed by Schwarzer and Jerusalem (1992); and validated in more than 30 languages (Luszczynska et al. 2005). The respondents assessed each of 10 statements related to coping in life. A high score, on the GSE scale, indicated high self-esteem; self-acceptance; and an optimistic attitude. In our sample, the GSE scale was unidimensional, and the Cronbach’s alpha reliability coefficient was equal to 0.842.

The second-dependent variable was the KIDSCREEN-10 health-related quality of life index. It was used, in the literature, as an abbreviated 10-item version of the full KIDSCREEN-52 questionnaire (Ravens-Sieberer et al. 2010). In our sample, KIDSCREEN-10’s reliability was equal to 0.774. The sum score was converted into a 0–100 scale, with a low score indicating a poor quality of life—feeling unhappy; unfit; and dissatisfied as regards family life; peers; and school life.

### Independent variables

The main independent SES variables were the mother’s education and family affluence. Data, from early childhood (1995 or 1998) and adolescence (2008), were considered and the change between these two periods was determined. Education was recoded in three comparable categories: below secondary; secondary (including post-secondary courses); and higher (tertiary undergraduate or postgraduate) education. A dichotomous variable, measuring change, was created, and a value of “1” was assigned to mothers who had increased their education level. In the models, taking into account only data from year 2008, six original categories of education were maintained: primary; basic vocational; secondary; post-secondary; BA (bachelor’s) degree; and higher than BA degree.

The analysis, of changes in family affluence, was more difficult due to the lack of comparable items. In 1998, only one question was available concerning retrospective subjective assessment of family living conditions in the first year of the child’s life (1995). Family conditions were classified in the following four categories as: very bad (found only in one family) or bad; average; good; and very good. In 2008, similar categories of family conditions were distinguished based on the modified FAS (Family Affluence Scale). Originally, FAS was composed of four items related to the adolescent having his/her own room; the number of computers and cars in the family; and holiday trips with the family outside the place of residence (Currie et al. 2008). After the fifth item, measuring subjective family wealth, assessed from the parent’s perspective, was added, the reliability, of this scale, rose from 0.49 to 0.58. A modified standardized index of affluence was estimated using the principal component’s method; this improved its continuity. Families, surveyed in 2008, were divided into four groups, proportionate to the earlier stage of research. These groups were labelled arbitrarily in the same manner as in 1995. Rather than illustrate the absolute level of family poverty/affluence, this division showed their relative position in the sample under consideration. There were five categories of family, whereby affluence, in the studied period, was described as: fell significantly (a difference of −3 or −2); fell slightly (−1); stayed the same (0); increased slightly (+1); or increased significantly (+2 or more).

An attempt was made, also, to include other available confounding variables: mother’s age (2008); her marital status (1995); the father’s education (2008); and family structure (2008). Because no significant relationship was noted, the use of these factors was discontinued.

### Statistical methods

Before all analyses, missing values were imputed by conditional mean procedure (Barzi and Woodward 2004), if applicable. Changes, in the level of education, were tested using the McNemar–Bowker method, i.e. the Chi-squared test for dependent variables. Mean GSE scores were compared using the one-factor ANOVA and the post hoc Scheffe’s test. A series of linear regression models was estimated whereby GSE was the dependent variable. A model, specified on the basis of the automatic selection procedure, was compared with: (1) the full model including all the factors; (2) the model using only the 2008 data; and (3) the model including only variables related to the changes between early childhood and adolescence. The standardized regression coefficients and the R-squared coefficient of determination were shown.

In the final part of the study, a potential further use, of all the obtained results, was discussed. The structural equation (path) model was assessed to evaluate whether self-efficacy could be the mediator of the relationship between a change in the mother’s education/family affluence and general well-being (KIDSCREEN-10). The effect of mediation was checked using the Baron and Kenny criterion (1986) and Sobel’s test.

The calculations were carried out using the PASW Statistics v. 17.0 and AMOS v.20 software.

## Results

### Changes in family socio-economic status between 1998 and 2008

Over the 10 years period, there was observed a significant increase (*p* < 0.001) in the level of the mothers’ education. The fraction of mothers, with university education, rose two-fold. In general, 12.6 % of mothers raised their level of education (8.6 % from secondary to tertiary education).

It was difficult to compare, between the two study periods, the absolute level of well off. However, using a similar distribution of the sample, it was found that only 44.5 % of the families were assigned, all the time, to the same category of wealth and, therefore, had not changed their position in relation to other families. It ought to be emphasized that a change, in the mother’s education, was linked to higher family affluence. Positive changes, in relative affluence, were recorded in 26.8 % of the families, in which the mother’s education level did not change, and in 37.3 % of families where a change occurred. In 2008, the mean standardized index of family affluence was −0.065 (SD = 0.99) and 0.515 (SD = 0.95) in those groups, respectively, *p* < 0.001.

### Univariate analysis of GSE determinants

Table 1 gives the mean GSE scores with regard to gender and SES variables with comparative response categories at two points of time. On a basis of post hoc Scheffe’s test on the 2008 data, a significant difference was noted between the children of the mothers with lower and higher than secondary education (*p* = 0.029) and when secondary and higher than secondary education were compared (*p* = 0.046). When considering the impact of affluence in 2008, the highest difference occurred between families living in poor and good conditions (*p* = 0.019).

**Table 1:** Mean general self-efficacy (GSE) scores in 13-year-old Polish adolescents according to gender, socio-economic factors and their change from childhood to adolescence (1995–2008)

Independent variables	*N*	%	Standardized (0–100) GSE score					
			Total		Girls		Boys	
			Mean	SD	Mean	SD	Mean	SD
Total	600	100.00	68.69	14.27	69.95	13.91	67.38	14.69
Mother’s education (1998)								
Lower than secondary	267	44.5	67.92	14.50	68.95	14.56	66.94	14.44
Secondary	275	45.8	69.18	13.76	70.26	13.05	67.91	14.51
Higher	58	9.7	69.83	15.57	73.33	15.27	67.17	15.48
*p*			0.484		0.330		0.863	
Mother’s education (2008)								
Lower than secondary	246	41.3	67.89	14.59	68.56	14.67	67.23	14.55
Secondary	241	40.4	68.18	13.46	69.27	12.79	66.90	14.17
Higher	109	18.3	72.23	14.47	74.78	14.10	69.82	14.53
*p*			**0.020**		**0.019**		0.434	
Change in mother’s education (1998–2008)								
No change	521	87.4	68.14	14.32	69.23	13.96	67.03	14.61
Higher than before	75	12.6	73.38	12.48	74.55	12.93	71.96	11.95
*p*			**0.003**		**0.023**		0.060	
Material conditions (1st year of child life)								
Poor or very poor	61	10.3	69.29	14.52	70.32	13.70	68.22	15.48
Average	304	51.2	67.92	14.95	68.94	13.98	66.83	15.89
Good	197	33.1	69.69	12.74	71.03	13.93	68.14	11.08
Very good	32	5.4	68.70	16.32	72.92	13.50	67.29	17.18
*p*			0.580		0.605		0.908	
Material conditions (2008)								
Poor or very poor	62	10.4	64.14	18.67	67.86	15.53	61.08	20.63
Average	301	50.6	67.95	13.88	68.58	13.98	67.37	13.78
Good	193	32.4	70.68	12.78	73.41	12.29	67.85	12.74
Very good	39	6.6	71.45	14.61	67.14	15.57	76.48	11.90
*p*			**0.006**		**0.029**		**0.004**	
Change in material conditions (1995–2008)								
Much worse	15	2.5	63.72	17.39	62.86	13.93	64.48	21.91
Worse	148	25.1	67.75	15.15	70.27	14.80	65.67	15.22
No change	262	44.5	68.08	14.22	69.38	13.48	66.60	14.95
Better	137	23.3	70.63	12.61	71.52	13.52	69.70	11.61
Much better	27	4.6	72.22	15.21	69.17	15.08	76.67	14.98
*p*			0.130		0.552		0.094	

Estimates in bold are statistically significant according to ANOVA test

**Fig. 2:**
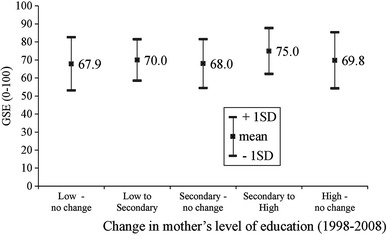
Mean general self-efficacy (GSE) score in 13-year-old Polish adolescents according to the change in the level of mother’s education (1998–2008)

There was found, also, to be a significant impact of a change in the mother’s education level. When comparing groups, with and without a change in the mother’s education, the average GSE scores were 73.4 and 68.1, respectively, *p* = 0.003. Comparing groups, with the same mother education level in 2008, the higher GSE score was found if the mother started from a relatively lower level of education (Fig. 2).

The difference in the mean GSE values, in the groups of families with extreme changes, i.e. when affluence rose and fell sharply, exceeded eight points; however, this result was not statistically significant (*p* = 0.130). This could be due to the small size of the extreme groups and the observation of a great variability of results in these groups. In general, in the group of families where affluence had not improved, the mean GSE was 67.9 (SD = 14.66) as opposed to 70.9 (SD = 13.04) in the group where an increase was noted (*p* = 0.019).

When comparing the mean GSE indexes, calculated for boys and girls, a generally higher impact of the mother’s education could be observed in girls.

### Multivariate analysis

A series of alternative regression models, adjusted for gender, were specified and estimated (Table 2). When all potential explanatory variables were added to Model 1, only the change, in the mother’s education level, and the modified FAS index were significant. In Model 2, only the 2008 data were used and a significant impact of current affluence; however, nothing of the mother’s current education was noted. In Model 3, only the effects of changes, in mother’s education and in family wealth, were considered and only the first parameter was significant. In Model 4, suggested by the method of the automatic selection of variables, the GSE was explained by a change in the mother’s education and family’s current wealth. These factors explained the GSE variability in 3.0 %, slightly better than in Models 2 or 3. Standardized parameters were higher in Model 4 than in Model 1; this suggested interaction between mother’s education and family affluence (both measured in 2008) as predictors of GSE.

**Table 2:**
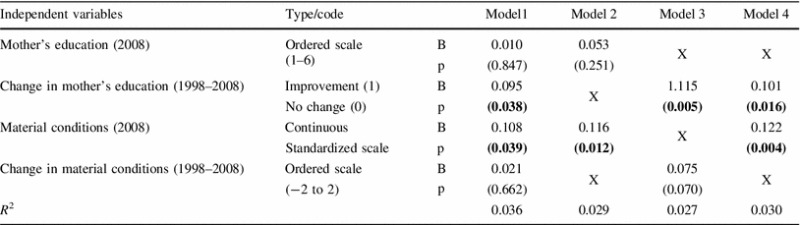
Socio-economic predictors of general self-efficacy (GSE) score in 13-year-old Polish adolescents—comparisons of models including and not including life-course perspective

Mother’s education coded from primary to higher than BA degree; changes in material conditions coded from much worse to much better

*B* unstandardized coefficient of linear regression adjusted for gender; *p,* *t* test for *B* (significant results in bold); *R* ^2^ coefficient of determination

### Example of the application of the research results in further studies

**Fig. 3:**
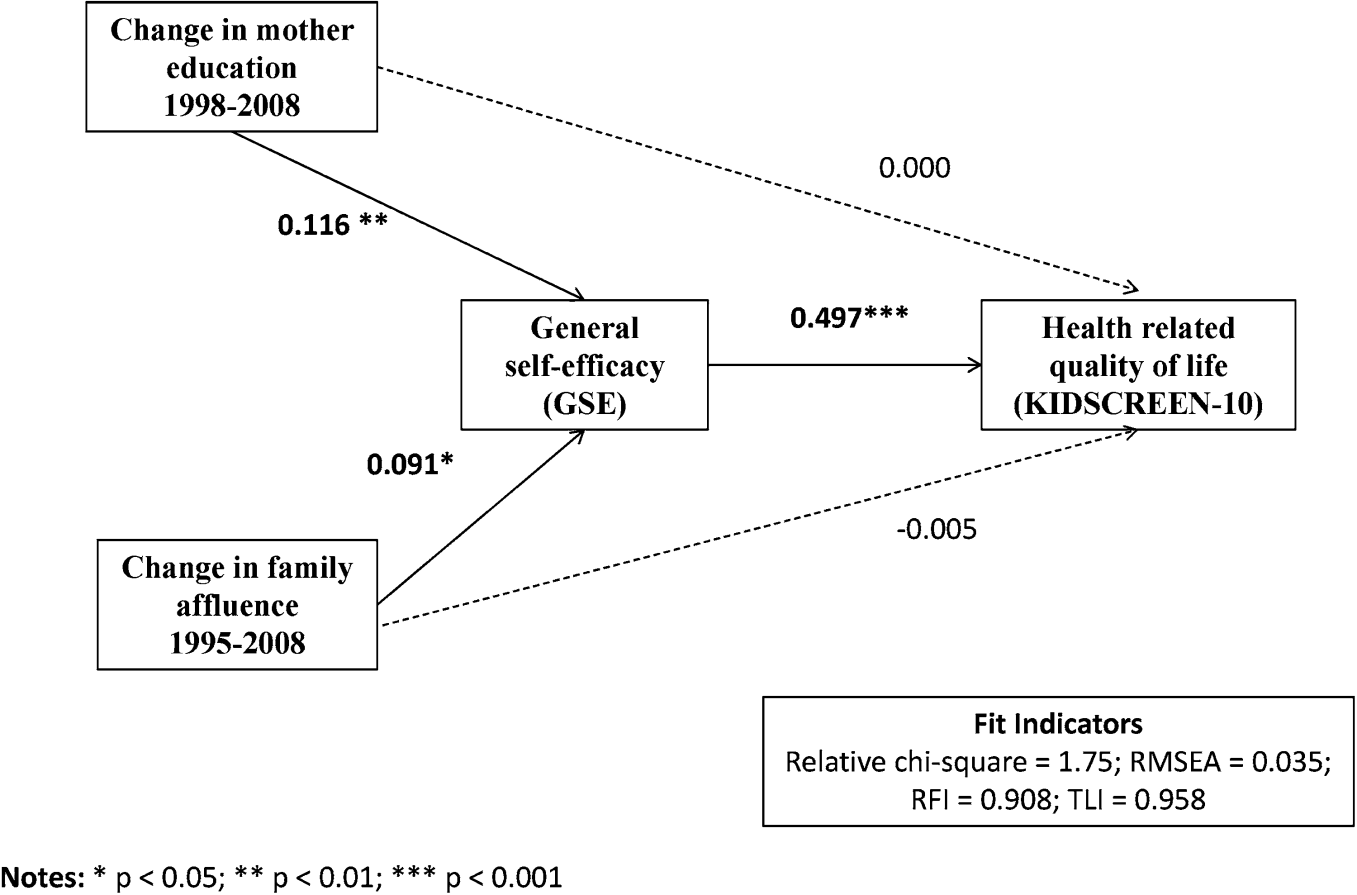
Path model representing direct and indirect effects of changes in family socio- economic status (Poland, 1995–2008) on health-related quality of life. Numbers on paths represent standardized regression weights

When considering that self-efficacy was an important determinant of the proper functioning of adolescents, and the obtained results could be used in devising more complex models featuring various outcome measures. A simple path model, whereby self-efficacy was the mediator of the relationship, between a change in family SES (both mother’s education and affluence) and well-being, could serve as an example. Although an increase, in the mother’s education and family affluence, resulted in a significant GSE increase, only a slight tendency for growth was present with regard to the KIDSCREEN-10 index. A strong correlation was found between the quality of life index and self-efficacy (*r* = 0.497; *p* < 0.001). The path model was estimated including direct and indirect relationship paths (Fig. 3). The Sobel’s test confirmed a significant mediation effect arising from a change in the mother’s education (*p* = 0.005), but not from a change in family affluence (*p* = 0.397). In addition, some goodness of fit statistics improved after the path of direct relationships was eliminated. For example, the RMSEA value fell from 0.084 to 0.035, and the upper limit, of the confidence interval, decreased to the recommended level of less than 0.08. The CMIN/DF value fell from 5.23 to 1.75. In the model, including direct paths, the RFI index was much worse (0.724 vs. 0.908), as was the TLI index (0.764 vs. 0.958).

## Discussion

This study’s main aim was to assess general self-efficacy of Polish 13 years olds in the context of their early childhood socioeconomic conditions. The mean level of the standardised GSE index, measured on a 0–100 scale, was 68.7. In this respect, the result, of the Polish study, was similar to the results obtained in other countries. In a Norwegian sample, the mean GSE score was 67.7 in an age-comparable group of adolescents (Kvarme et al. 2009).

Our study had two main new findings: first, we could show that improving mother’s education could affect the development of resources important for adolescent health. Secondly, these resources could serve as mediators in the models of early childhood social determinants of health.

We attempted to evaluate whether the current education of the mother and family affluence or the changes, which occurred with regard to these indicators, were strong predictors of self-efficacy at age 13. According to Bandura’s social learning theory (Bandura 1976), vicarious experiences are one of the main sources of self-efficacy. A lot of new behaviours were acquired through the observation of other people’s behaviours. The parents’ ability to cope with a new challenge, for example, raising the education level, created an opportunity for the children to observe their behaviours. Other studies proved that women, who attempted to raise their qualifications had higher self-efficacy; in the future, this might result in higher self-efficacy of their children (Chang 2003).

A series of regression models were compared. In the best one, GSE was explained by the change in mother’s education and family’s current affluence. It ought to be pointed out that, where it was included, the mother’s raised education level was a significant factor in all alternative models. Most research focused rather on the impact of the absolute education level than on its relative changes. Moreover, in longitudinal studies, it was much easier to follow changes in education rather than changes in family wealth.

Previous studies, on parental influence on a child’s assets, were carried out with regard to adolescents who were asked to assess parental support; parental control; and their parents’ resources of knowledge (Frank et al. 2010; Graziano et al. 2009). In some studies, there was a particular focus was on specific self-efficacy related to a given skill. Studies, relating to the dietary self-efficacy of adolescents, found that parental knowledge was an important factor in improving healthy eating in adolescents (Pearson et al. 2012). The example, presented in the final part of our paper, indicated that self-efficacy might be a mediator of the relationship between a parent’s raised education level and other variables related to general well-being. A similar effect could be achieved by examining the influence of other personal assets on various health outcomes; this was consistent with the results of other studies (Leganger et al. 2000).

### Strengths and limitations

Because our study was an analysis of secondary data, it had some limitations. The first two stages of this three-wave study were designed for a different purpose than the last one. In addition, they were designed, even less so, to study the influence of changes in family socioeconomic status on selected internal resources in adolescence. Moreover, a limited number of variables describing a changing family situation was applied. Despite the attempts to extend the set of confounding variables, no predictor of deterioration, in GSE, was identified. Similar to the National Longitudinal Survey of Youth (Ryan and Claessens 2012) we could consider only additional data on mother marital status, at the time of the child’s birth, and on family structure in early adolescence. Having two points of time did not allow us to establish when the change, in family status, occurred. In particular, it was difficult to evaluate the effect of negative changes, such as divorce. If over time, single-parent and blended families become more common, parents and children might perceive changes, in these structures, as more normal, more predictable, and, therefore, less stressful. Finally, no information was collected about parent self-efficacy (in particular, the mother’s self-efficacy). It was known, from other cited studies, that mothers, with a high level of self-efficacy, continued to study more frequently. In adolescents, the determinants of self-efficacy including school and peer environment, were definitely more complex (Caprara et al. 2002). This kind of research ought be carried out on a larger sample; this would allow for a multi-factor analysis. In considering the large size of the primary sample at the first stage, only a fraction of the cases were included in the final analysis. This was a consequence of including only 20 % of the initial sample at the second stage.

Nevertheless, our study had a number of advantages which, we hoped, would counterbalance the presented shortcomings. Currently, in Central and Eastern Europe, studies, which are based mostly on a cross-sectional design, are being conducted on changes in social gradient of health and health behaviours (Mazur and Woynarowska 2011; Pitel et al. 2012). Cohort studies, such as presented herein, were recognized as the best sources of data in linking early childhood events with later outcomes (Robert 1997). One advantage of the study was its significant territorial diversity; usually, this was not achieved in studies of adolescents carried out in schools. Another advantage included the use of a coherent age group and, therefore, eliminating an additional age factor.

The use of the FAS Scale, as a measure of material conditions, could be questioned. This scale was used widely in other papers (e.g. Holstein et al. 2009), but some authors criticised its low internal consistency (Molcho et al. 2007). The modification of the original FAS, improved its reliability, but not sufficiently. However, the results of validation studies, conducted, also, in Poland in 2003 (Mazur 2010) and 2005 (Andersen et al. 2008), could serve as an argument in favour of using it. These studies showed high compliance of child–parent responses and strong correlation between the overall FAS index and external income measures. The original method of analysing a change in family affluence was devised; this increased data comparability. This method was the authors’ adaptation of an approach, used in research of households, based on income distribution (quantiles). To our knowledge, this study is one of few studies describing that kind of relationship on the basis of prospective design.

### Implications for policy and further research

Our study belongs to a stream of research relating to social health inequalities from the life course perspective. It referred to practical measures, in particular those related to the strategy of lifelong learning and creating a home learning environment (HLE). In contrast to other studies, the focus was not on the negative impact of low social position but on the positive influence of raising it. Attention was brought to the fact that the transition from late childhood to adolescence occurred at a time when families underwent a series of economic transformations in their aspiration to improve living conditions and to achieve stable affluence. According to Delors’ report (Delors 1996), lifelong learning should encompass many activities enabling people to familiarize themselves with the phenomena of the changing world; social changes; and, above all, themselves. Raising parental education, apart from having direct benefits for themselves and for society development, was very important, also in addition, for their children. Knowledge and skills, acquired by the parents, might support a harmonious functioning of families and normal child development. It might be assumed, that parents, themselves interested in further education, would create a positive home learning environment. Currently, HLE creation was considered to be one of the strategies for reducing poverty and social inequalities in health.

The obtained results might serve as an inspiration to undertake research concerning the impact of changes in socioeconomic status on other developmental assets conducive to adolescent health.
